# How do *K*-*RAS*-activated cells evade cellular defense mechanisms?

**DOI:** 10.1038/onc.2015.153

**Published:** 2015-05-11

**Authors:** Y-S Lee, S-C Bae

**Affiliations:** 1 Department of Biochemistry, School of Medicine, and Institute for Tumor Research, Chungbuk National University, Cheongju, South Korea

## Abstract

Lung adenocarcinomas, like other cancers, develop through the accumulation of epigenetic and genetic alterations. Numerous studies have shown that *K-RAS* mutation is among the most important early events in carcinogenesis of the lung. However, it is also well established that growth-stimulating signals feed back into growth-suppressing pathways, and any imbalance in these signaling networks will cause the cell to exit the cell cycle, thereby preventing uncontrolled cell growth. How, then, do *K-RAS*-activated cells evade cellular defense mechanisms? To answer this question, it is necessary to identify the molecular event(s) responsible for the development of early dysplastic lesions that are unable to defend against aberrant oncogene activation. Lineage-determining transcriptional regulators govern differentiation status during normal lung development, as well as in lung adenocarcinoma. Among the genes involved in *K-RAS*-induced lung tumorigenesis, *RUNX3* is unique: inactivation of *Runx3* in mouse lung induces lung adenoma and abrogates the ARF–p53 pathway. This observation raises the possibility of intimate cross-talk between the differentiation program and oncogene surveillance. In this review, we summarized evidences suggesting that *K-RAS-*activated cells do not evade cellular defense mechanisms *per se*; instead, cells with *K-RAS* mutations are selected only if they occur in cells in which defense mechanism is abrogated.

## Introduction

Lung cancer is one of the most commonly diagnosed malignancies worldwide and the leading cause of cancer-related death.^1, 2^ Lung cancers comprise four histological types as following: adenocarcinoma, squamous cell carcinoma, large cell carcinoma and small cell carcinoma. Of these four types, adenocarcinoma is the most common and its frequency is increasing rapidly.^3, 4^

**Figure 1:**
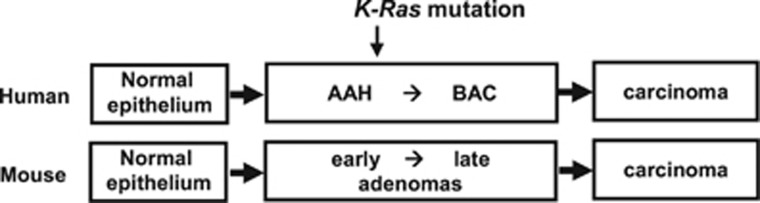
Current paradigm of lung adenocarcinoma. The majority of lung adenocarcinomas develop through a multi-step tumorigenesis pathway. Tumors develop from atypical adenomatous hyperplasia (AAH) to bronchioalveolar carcinoma (BAC), and ultimately progress to multiple types of invasive tumors. Mouse lung adenoma is equivalent to human AAH and BAC. RUNX3 is inactivated in most early AAH (human) and early adenoma (mouse). *K-Ras* activation is detected in relatively late AAH or adenoma.^21, 48^

According to the current paradigm, lung adenocarcinoma develops primarily by step-wise progression accompanied by sequential morphologic changes, from atypical adenomatous hyperplasia (AAH) to bronchioloalveolar carcinoma (BAC), and finally to multiple types of invasive tumors, including mucinous and non-mucinous adenocarcinomas.^5^ AAH/BAC can be divided into two major subtypes, mucinous and non-mucinous;^6^ *K-RAS* is predominantly mutated in mucinous AAH/BAC.^7^ Mouse lung adenoma is considered to be equivalent to human AAH/BAC (Figure 1).

The progression of lung adenocarcinomas from adenomatous growth to carcinomas proceeds essentially according to the multi-step tumorigenesis pathway established in colon cancer.^8, 9^ However, the key genetic and epigenetic alterations that are responsible for clonal expansion following each step of tumorigenesis are less well established for lung cancer than for colon cancer. In particular, the critical initial molecular events in the development of lung adenoma remain unclear. Numerous studies have reported that *K-RAS* mutation, the genetic alteration most frequently detected in lung cancer, is an early event responsible for the development of lung adenoma.^10, 11, 12, 13, 14^ However, the p14^ARF^–p53 pathway effectively defends the cell against aberrant oncogene activation.^15, 16, 17^ Therefore, it is important to determine whether one or more hidden tumor suppressors are inactivated in lung adenomas, or instead the p14^ARF^–p53 pathway simply provides an incomplete defense against oncogenic *K-RAS*-induced tumorigenesis. If such hidden tumor suppressors exist, this issue could be resolved by identifying them. Inactivation of key tumor-suppressor genes is often caused by epigenetic process; unfortunately, however, identification of epigenetically silenced tumor suppressors is particularly difficult because gene silencing also occurs over the course of normal differentiation. Therefore, to understand how specific changes in genotype collaborate to create the cancer phenotype, we must actively intervene in the process of tumorigenesis in animal models.

Recent studies aimed at identifying genes involved in the development of lung adenocarcinomas have revealed important roles for lineage-determining transcription factors in *K-RAS*-induced lung tumorigenesis.^18, 19, 20^ Inactivation of these genes markedly promotes progression of *K-Ras-*induced mouse lung adenoma into adenocarcinoma. However, inactivation of most lineage-determining transcription factors does not induce adenoma; the notable exception is *Runx3*. In mouse lung, inactivation of *Runx3* induces adenoma and abrogates oncogene surveillance mechanisms.^21^ These observations provide an important clue about how adenoma develops and how *K-RAS*-activated adenoma cells evade cellular defense mechanisms.

## Lung epithelial cells

Genetic and epigenetic lesions have major roles in determining tumor phenotypes. However, cancers with distinct phenotypes may also originate from different types of cells within an organ. Therefore, the various distinct types of lung adenocarcinomas can develop either as a result of different molecular events or because they originate from different cell types.^22, 23, 24^

**Figure 2:**
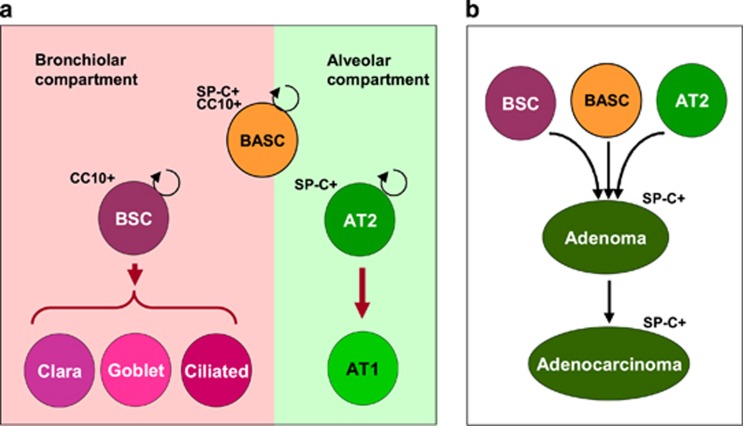
Lung epithelial cells. (**a**) Bronchioalveolar stem cells (BASCs) give rise to bronchiolar epithelial cells (BSC or Clara cells) and alveolar epithelial cells (ASC or AT2 cells), which are involved in tissue renewal. BASCs express both BSC-specific markers (*CC10*) and ASC-specific markers (*SP-C*). BSCs generate BSCs, Clara cells, goblet cells and ciliated cells, whereas AT2 cells generate AT2 and AT1 cells. (**b**) BASCs, BSCs and AT2 cells are the known cells of origins of lung adenomas/adenocarcinomas.

The lung has two distinct cellular compartments, the alveolar and bronchiolar epithelium. The alveolar epithelium includes alveolar epithelial type 1 (AT1) and AT2 cells, which are derived from AT2 cells. The bronchiolar epithelium includes basal cells, ciliated cells, Clara cells, goblet cells and a few pulmonary neuroendocrine cells.^25, 26, 27, 28^ At least three types of progenitor cells have key roles in maintaining the bronchiolar epithelium of the lung: (1) basal cells, which generate Clara cells and ciliated cells; (2) variant Clara cells (also called bronchiolar stem cells, BSCs), which also generate Clara cells, goblet cells and bronchiolar neuroendocrine cells; and (3) bronchioalveolar stem cells (BASCs) (Figure 2a). BASCs are characterized by their specific location at bronchiolar–alveolar duct junctions and by the expression of both a Clara cell-specific marker (*CC10*, also known as *SCGB1A1* or *CCSP*) and an AT2 cell-specific marker (*SP-C*).^11^ All types of progenitor cells can develop into lung adenocarcinoma in an oncogenic *K-Ras-*induced mouse lung cancer model^24, 29, 30, 31^(Figure 2b).

## *K-RAS* mutation is critical for the development of lung adenocarcinoma

*RAS* genes were originally discovered in studies of cancer-causing retroviruses in animals. Two rat sarcoma (RAS)-inducing viruses, Harvey's *Ras* (*H-ras*) and Kirsten's *Ras* (*K-ras*), were identified in the 1960s.^32, 33^ Subsequently, transforming genes were isolated from human cancer cell lines in the early 1980s. These genes turned out to be the human homologs of rat *H-ras* and *K-ras.*^34, 35, 36^ A third ras gene (*N-ras*) was discovered in human cancer cells at 1983.^37, 38^ *RAS* family genes encode a membrane-bound GTP-binding protein that transmits extracellular stimuli through receptor tyrosine kinases. Single point mutations, most of which occur at codon 12, 13 or 61 in RAS genes, result in constitutive activation of the encoded proteins.^39, 40^

*K-RAS* is one of the most frequently mutated oncogenes in lung adenocarcinoma patients. Approximately 76% of mucinous lung adenocarcinomas have *K-RAS* mutations, but non-mucinous type adenocarcinomas rarely have the mutation. On the basis of this observation, the role of oncogenic *K-RAS* in lung cancer initiation has been extensively studied using various knock-in mouse models. Mice of the *K-Ras*^*LA*^ strain, in which expression of *K-Ras*^*G12D*^ can be spontaneously activated by random recombination, develop lung adenomas.^12^ Similarly, in a *LoxP-Stop-LoxP-K-Ras* conditional mouse strain (*K-Ras*^*LSL-G12D*^), expression of oncogenic *K-Ras* is controlled by a removable transcriptional termination *Stop* element; when the expression of endogenous level of *K-Ras*^*G12D*^ was triggered in lung by *Adeno-Cre*-mediated deletion of the *Stop* sequence, the mice developed adenomas in a month of stimulation.^10, 41^

Although these experiments demonstrated the critical role of oncogenic *K-RAS* in lung tumorigenesis, it remains unclear whether all *K-RAS*-activated cells are tumorigenic, or instead only some cells are susceptible to *K-RAS*-induced tumorigenesis. To address this question, Guerra *et al.* generated *K-Ras*^*LSL-G12D*^*-IRES-geo* mice harboring a *K-Ras*^*LSL-G12D*^ allele containing *IRES-geo*, which enables tracing of targeted cells; they then targeted *K-Ras*^*LSL-G12D*^*-IRES-geo* throughout the whole body with tamoxifen-inducible Cre-ERT2 and analyzed the effect on tumor development.^42^ Notably, systematic tamoxifen treatment induced *K-Ras*^*LSL-G12D*^*-IRES-geo* in 5–15% of cells in most tissues, which include stem cells. However, expression of *K-Ras*^*G12D*^ throughout the body failed to induce unscheduled proliferation or other growth abnormalities for up to 8 months. Only a subset of *K-Ras*^*G12D*^-expressing lung epithelial cells underwent malignant transformation several months after inducer treatment. These results suggested that only a very small number of cells in a specific cellular context are transformed by oncogenic *K-Ras.*^42^

In that case, what kinds of cells are susceptible to oncogenic *K-Ras*-induced tumorigenesis? Kim *et al.*^11^ reported that rare BASCs, which express both *SP-C* and *CC10*, are the source of oncogenic *K-Ras*-induced lung adenocarcinomas. Xu *et al.*^43^ showed that expression of *K-Ras*^*G12D*^ in AT2 cells (*SP-C*-expressing cells) leads to adenocarcinoma formation, suggesting that oncogenic *K-Ras*-expressing AT2 cells develop into adenocarcinomas. Cho *et al.*^29^ reported that constitutive expression of *K-Ras*^*G12D*^ in Clara cells (that is, *CC10*-expressing cells) led to adenocarcinoma, suggesting that lung adenocarcinoma could also originate in bronchiolar epithelial cells. These results demonstrate that normal lung epithelial cells, BASCs, Clara cells and AT2 cells can develop into lung adenocarcinomas upon *K-Ras* activation (Figure 2).

## *K-RAS* mutation alone does not seem to initiate lung tumorigenesis

Notably, Clara and AT2 cells are relatively abundant in lung epithelium, inconsistent with previous observations that only a very limited number of lung epithelial cells are susceptible to oncogenic *K-Ras-*induced lung tumorigenesis.^44^ In addition, a simple calculation based on the rate of specific point mutations per cell and the number of cells in the body suggests that several thousand new point-mutated *RAS* oncogenes are created every day in every human being.^7^ However, humans do not suffer from cancer as frequently as this calculated rate would predict. Even Costello syndrome patients, who carry *H-RAS*^*G12A*^ mutant alleles in their germ lines, do not develop tumors at young age. Only 24% of Costello syndrome patients develop malignancy, and then only after several decades of life.^45^ Therefore, it is likely that normal cells are resistant to transformation by *RAS* activation alone, and that other genetic or epigenetic alterations are also required. This idea is supported by the observation that *K-RAS* mutation is a relatively late event in the pathogenesis of human lung adenocarcinoma: *K-RAS* mutations appear to be involved in the conversion of dysplastic cells to preinvasive cancer cells, rather than initiation of preneoplastic lesions.^46^

## Dysregulation of the differentiation program in lung tumorigenesis

What kind of genetic or epigenetic alterations are involved in the oncogenic *K-RAS*-induced lung tumorigenesis? It is worth noting that mouse lung adenocarcinomas induced by oncogenic *K-Ras* alone are all of the non-mucinous type, regardless of the cell type of origin. In humans, however, *K-RAS* mutation is far more frequent in mucinous than non-mucinous lung adenocarcinomas. Because these subtypes of lung adenocarcinomas are distinguished by the differentiation status of the tumors, we imagine the involvement of differentiation regulators in *K-Ras*-induced lung tumorigenesis. Development of lung adenocarcinoma is often associated with dysregulation of lung epithelial lineage-determining transcriptional regulators that govern differentiation status.^47^ For example, *Gata6* maintains proper alveolar maturation^18^ in cooperation with other known lineage-specific transcription factors such as *Hopx*^19^ and *Nkx2-1.*^20^ *Runx3* is required for both bronchiolar and alveolar lineage differentiation.^48, 49^ Among the differentiation regulators, the roles of *Nkx2-1* and *Runx3* in oncogenic *K-Ras*-induced lung tumorigenesis have been most extensively studied.

*Nkx2-1* (also called *Titf1* or *Ttf-1*), which is essential for lung epithelial lineage determination, is frequently up- or downregulated in poorly differentiated lung adenocarcinomas.^50, 51^ Winslow *et al.*^52^ noticed that *Nkx2-1* is frequently silenced in malignant adenocarcinomas in a *Kras*^*LSL-G12D*^*;p53*^*−/−*^ mouse cancer model. Although *Nkx2-1*^*+/–*^ mice do not develop spontaneous lung tumors, overexpression of *K-Ras*^*G12D*^ in *Nkx2-1*^*+/–*^ mouse lung results in a larger number of malignant lung tumors (with greater volumes) than in wild-type mice.^53^ Snyder *et al.*^54^ also demonstrated that simultaneous *Kras*^*G12D*^ expression and *Nkx2-1* deletion in lungs (*Kras*^*LSL-G12D*^*;Nkx2-1*^*−/−*^) results in early onset malignant adenocarcinoma. Notably, simultaneous *Kras*^*G12D*^ expression and *Nkx2-1* inactivation induces mucinous-type lung adenocarcinomas, whereas *Kras*^*G12D*^ expression alone induces only non-mucinous type lung adenoma/adenocarcinomas. To determine whether *Nkx2-1* inactivation occurs earlier than *K-Ras* activation, Snyder *et al.*^54^ inactivated *Nkx2-1* in established *Kras*^*LSL-G12D*^-induced tumors and showed that non-mucinous-type tumor cells produced mucin upon *Nkx2-1* inactivation. However, deletion of *Nkx2-1* in adult lung does not induce adenoma.^53, 54^ Therefore, inactivation of *Nkx2-1* appears to be involved not only in tumor initiation but also in the transition from adenoma to mucinous adenocarcinoma, although deletion of *Nkx2-1* alone does not result in the development of adenoma.^54^

*RUNX3*, a lineage-determining transcription factor expressed in multiple tissues, is frequently downregulated in various tumors.^45, 49^ During lung development, *RUNX3* has an essential role in terminal differentiation of lung epithelial cells: it is required for the generation of bronchiolar lineage cells and for the terminal differentiation of alveolar cells.^45^ Lee *et al.*^48^ found that *RUNX3* is inactivated in nearly all human lung AAH and carcinogen-induced early mouse lung adenomas in which *K-Ras* is not yet mutated. Subsequently, they investigated whether *Runx3* inactivation is causally associated with the development of adenomas. Surprisingly, deletion of *Runx3* in adult mouse lung results in the early induction of lung adenomas of either the mucinous or non-mucinous type.^21^ Simultaneous targeting of *Runx3*^*f/f*^ and *K-Ras*^*LSL-G12D/+*^ led to the progression of the two distinct types of adenomas into the corresponding types of adenocarcinomas. Therefore, *Runx3*^*f/f*^ and *Runx3*^*f/f*^*;K-Ras*^*LSL-G12D/+*^ mice faithfully recapitulate the development of human lung AAH and adenocarcinoma, respectively.^21^ Although the specific cells of origin of each type of adenoma remain to be identified, these results clearly demonstrate that inactivation of *Runx3* is responsible for the development of multiple types of lung adenomas.

If *K-Ras* activation alone does not induce lung adenoma, but *Runx3* inactivation does, how can lung adenoma develop in a mouse model in which *K-Ras* is activated alone? *Runx3* is expressed in nearly all lung epithelial cells, but silenced in most *Kras*^*G12D*^-induced lung adenocarcinomas,^21^ implying that *Kras*^*G12D*^ can induce adenomas when it is expressed in cells that do not express *Runx3* or some other critical gene. These *Runx3* non-expressing cells could be rare normal cells or abnormal cells generated by deregulation of gene expression. The former possibility is supported by the observation that BASCs, which express both *SP-C* and *CC10*, are the targets of oncogenic *K-Ras-*induced lung tumorigenesis.^11^ On the other hand, the latter possibility is supported by the observation that a large proportion of *Runx3*-inactivated bronchiolar epithelial cells express both *SP-C* and *CC10*, and these cells develop into adenomas.^21^ Although both possibilities are supported by evidence, the latter is more plausible under physiological conditions, because tumor development is considered to be a biological process that resembles Darwinian evolution: random mutations create genetic variability in a cell population, and the force of selection favors the outgrowth of individual mutant cells that happen to be endowed with advantageous traits. On the basis of a combination of Darwinian theory and the concept of multi-step tumor progression, tumorigenesis is now understood as a succession of clonal expansions.^8, 9^ Selection of cells endowed with advantageous traits requires a large number of cells. However, single-step tumorigenesis by oncogenic *K-Ras* mutation from rare (but normally existing) cells is not consistent with the Darwinian concept. On the other hand, if *Runx3* were inactivated by chance in normal cells, these cells would acquire a proliferative advantage,^21^ enabling subsequent selection for *K-Ras-*mutated cells. Undoubtedly, *K-Ras-*induced lung adenocarcinoma development can proceed via multiple pathways; nonetheless, the high frequency of *Runx3* inactivation in *K-Ras-*induced mouse and human lung adenocarcinoma suggests that a major pathway involves *Runx3* inactivation before *K-Ras* activation.

## Critical role of *Runx3* in the defense against oncogene activation

Several important differentiation regulators govern lung development. However, deregulation of the differentiation program is not sufficient to induce adenoma: so far, *Runx3* is the only gene whose inactivation has been reported to induce lung adenoma. What makes *Runx3* is so special in regard to lung tumorigenesis?

**Figure 3:**
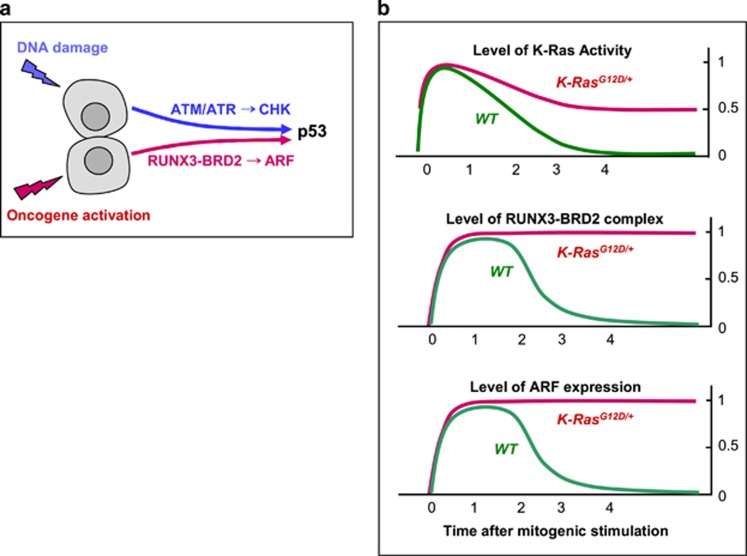
p53 tumor-suppressor pathways. (**a**) Two major pathways trigger p53 activation. (1) DNA damage stress is sensed by the ATM/ATR kinases, which activate the CHK1/CHK2 kinases, which in turn stabilize p53. Aberrant oncogene activation is sensed by the RUNX3–BRD2 complex, which induces expression of ARF, which in turn inactivates HDM2 and thereby stabilizes p53. (**b**) Mechanism for sensing constitutive RAS activation. Normal RAS activity is downregulated to the basal level soon after mitogenic stimulation (top panel, green line). Although RAS is activated, the RUNX3–BRD2 complex is formed (middle panel, green line) and ARF expression is induced (bottom panel, green line). In normal cells, RUNX3–BRD2 complex formation and ARF expression occurs for only a short time (1–3 h after mitogenic stimulation) and disappears when RAS activity is downregulated. However, heterozygous mutation of RAS results in maintenance of 50% of the maximum level of RAS activity. This persistent RAS activity maintains the RUNX3–BRD2 complex and ARF expression until the G_1_/S check point.

It is well established that cells have evolved effective defense mechanisms against cellular transformation. Ever since it became clear that about 50% of human cancers contain mutations in *p53*, this gene has been intensively studied as a cellular defense against transformation. The p53 transcriptional program includes the activation of number of pro-apoptotic proteins and cell cycle inhibitors, resulting in apoptosis or irreversible proliferative arrest.^55, 56^ Two major stresses, DNA damage and oncogene activation, trigger p53 activation through different genetic pathways: DNA damage through the ATM/ATR and CHK1/CHK2 kinases, and oncogenic signaling through p14^ARF^ (in mouse, p19^Arf^; hereafter, ARF or Arf)^57^ (Figure 3a). Recent genetic evidence in mice indicates that ARF-dependent activation of p53 is critical for p53-mediated tumor suppression.^58^

Hence, it is important to determine the role of the ARF–p53 pathway in oncogenic *K-RAS*-induced lung cancer. Indeed, simultaneous activation of oncogenic *K-Ras* and inactivation of the *p53* tumor suppressor in mouse lung significantly accelerates the malignancy of the resultant adenocarcinoma.^41^ However, it remained unclear whether inactivation of *p53* contributed to the initiation or progression of lung tumorigenesis. To address this issue, Junttila *et al.* and Feldser *et al.* induced lung adenocarcinoma by simultaneous inactivation of *p53* and *K-Ras* activation, and then restored *p53*. Importantly, restoration of p53 activity only resulted in the regression of adenocarcinoma and did not affect adenoma.^13, 14^ In addition, the Arf–p53 pathway was retained in mouse embryonic fibroblast cells expressing *K-Ras*^*G12D*^.^42, 59^ These results suggested that the p53 pathway is not engaged in the early stage of lung tumorigenesis, even if oncogenic *K-Ras* is expressed.

Why does the defense mechanism not prevent tumor formation in mice? Palmero *et al.*^60^ demonstrated that overexpression of oncogenic *K-Ras* activates the Arf–p53 pathway in primary cells. On the other hand, Junttila *et al.*^13^ and Feldser *et al.*^14^ showed that oncogenic *K-Ras* expressed at the endogenous level does not activate the Arf–p53 pathway in mouse lung. These observations might be explained in two major ways as follows: (1) the p53 pathway has an inherent limit and is not engaged by expression of an activated oncogene at the endogenous level that is sufficient to induce tumors or (2) the p53 pathway fails to be activated not as a result of some inherent limit but instead due to some unknown component(s) that mediates oncogenic activity. Although several lines of evidence support the first possibility,^13, 14^ several studies have reported that the activation of *RAS* alone in normal cells is not sufficient to induce transformation.^45, 46^

Therefore, we must consider the second possibility. ARF, which is induced in response to oncogenic activation, stabilizes p53 by inhibiting HDM2 (in mouse, MDM2).^61^ Mitogenic signaling activates the GTPase activity of RAS, which decreases to the basal level soon after the signal is transduced to downstream kinase pathways. Oncogenic RAS is a constitutively active form whose activity is not downregulated. Therefore, heterozygous *RAS* mutation results in maintenance of 50% of the maximum level of Ras activity (Figure 3b). To protect themselves from oncogenic *RAS*-induced abnormal proliferation, cells should be able to sense the duration of the 50% RAS activity rather than its maximum level of activity. For a long time, however, it was unclear whether cells can indeed recognize aberrant persistence of RAS activity. Lee *et al.*^21^ demonstrated that mammals have evolved an effective defense mechanism against the persistent activation of oncogenic RAS. When RAS is activated by normal mitogenic stimulation, RUNX3 forms a complex with p300 and BRD2 (a relative of TAF250) in a MAPK activity-dependent manner; this complex transiently induces *ARF*, which in turn transiently stabilizes p53. Soon after the mitogenic surge, MAPK activity is reduced. In this situation, the Runx3–BRD2 complex dissociates and *ARF* expression is repressed. Mitogen-stimulated transient activation of the ARF–p53 pathway does not affect the cell cycle because it occurs only 1–3 h after mitogenic simulation and is then silenced at the G_1_/S checkpoint. When *K-RAS* is constitutively activated, the RUNX3–BRD2 complex is maintained, and expression of *ARF* and *p53* continued until the G_1_/S checkpoint.^21^ These results show that cells can effectively defend against an endogenous level of RAS activity, and that the RUNX3–BRD2 complex functions as a sensor for abnormal persistence of RAS activity^21^ (Figure 3b).

**Figure 4:**
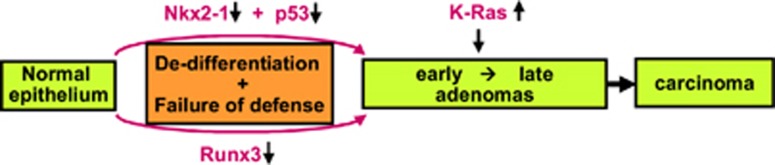
A model for the step-wise progression of lung adenocarcinoma. Adenoma development may require abrogation of both the differentiation program and oncogene surveillance. These two events could occur independently: for example, the differentiation program could be abrogated by silencing of *Nkx2-1*, and oncogene surveillance mechanism could be disabled by *p53* deletion. Alternatively, the two events could occur simultaneously by inactivation *Runx3*, allowing adenoma cells to progress to adenocarcinoma upon oncogenic *Ras* mutation.

These results demonstrate that *RUNX3* has essential roles in oncogene surveillance, as well as regulation of differentiation. To date, *RUNX3* is the only gene whose inactivation has been shown to be sufficient to induce adenoma, suggesting that abrogation of both the differentiation program and oncogene surveillance mechanism might be required for adenoma development. Such events could occur either as a result of multiple molecular events (that is, one involved in each pathway) or a single molecular event such as *RUNX3* inactivation. Cells acquired *K-RAS* mutation might be selected when it occurred in cells in which differentiation program and defense mechanism is abrogated (Figure 4). Obviously, the probability of deregulation is much higher for a single gene than for two genes. This might explain why *RUNX3* inactivation is so frequently detected in lung AAHs.^21^

## Prospects

It is somewhat surprising that a differentiation regulator, *RUNX3*, has a key role in oncogene surveillance. However, considering the machinery involved in cell cycle decision making, we can imagine the existence of contact points between the differentiation program, cell cycle progression and oncogene surveillance. When stimulated by mitogens, cells decide whether they will remain in G_1_, retreat from the active cycle into G_0_ or advance into the remaining phase of the cell cycle.^60^ This critical decision, which is dependent on cell context, is made 2–3 h after stimulation, at the so-called restriction point (R-point).^62, 63^ A growing body of evidence indicates that deregulation of the R-point decision-making machinery accompanies the formation of most if not all types of cancer.^7^ To make an appropriate decision, cells should know their own differentiation status, and *RUNX3* might serve to report cellular differentiation status to the R-point ‘committee'. Hence, it would be interesting to investigate whether the tumor-suppressor activity of *RUNX3* is associated with R-point commitment.

Although numerous anti-cancer drugs have been developed, most cancers are still ultimately fatal due to the very high rate of recurrence. For example, gefitinib effectively eliminates *EGFR*-mutated lung adenocarcinomas at the beginning of therapy, but the cancers recur in 90% of patients within 2 years.^64^ Recurrence of lung cancer occurs mainly due to persistent early lesions that are resistant to the anti-cancer drug. Therefore, to eradicate cancers, it is necessary to eliminate both early and malignant lesions. Because the critical genes involved in the initiation of adenoma development were unknown, to date no anti-cancer drug has been developed that is capable of eliminating early lesions. As noted above, a recent effort to restore *p53* in *K-Ras-*activated mouse tumors failed to eliminate adenoma: *p53* restoration killed only malignant adenocarcinomas, leaving adenomas untouched.^13, 14^ However, the recent findings that *RUNX3* is inactivated in most AAHs in human, and that *Runx3* inactivation in mice induces lung adenoma, implies that *RUNX3*-targeted therapies could be a means to eradicate adenomas. It would be exciting indeed if *Runx3* restoration in *K-Ras*-activated tumors is able to eliminate not only adenocarcinomas but also adenomas. If that turns out to be the case, *RUNX3* will be a promising target for curative cancer therapy.

Is it possible to reactivate *RUNX3* in tumors? It is worth emphasizing that *RUNX3* is inactivated in various human tumors, mainly by epigenetic alteration. In theory, epigenetically inactivated genes could be reactivated by DNA methyltransferase inhibitors or histone deacetylase (HDAC) inhibitors. Class III HDACs (as well as class I and II HDACs) inactivate *RUNX3*, and class III HDACs can be inhibited by physiological concentrations of niacinamide.^65, 66^ Indeed, inhibition of class III HDACs by niacinamide^65, 66^ reactivates or protects *RUNX3*, thereby effectively preventing carcinogen-induced tumorigenesis in the bladder,^67^ liver^68^ and lung (our unpublished observation). Because niacinamide is nontoxic, it could be used to prevent tumors. Therefore, identification of *RUNX3* as a potential specific target for early-stage lung cancer eradication provides an important theoretical basis for the development of safer and more effective anti-cancer drugs.
